# Prediction of Soil Deformation in Tunnelling Using Artificial Neural Networks

**DOI:** 10.1155/2016/6708183

**Published:** 2015-12-24

**Authors:** Jinxing Lai, Junling Qiu, Zhihua Feng, Jianxun Chen, Haobo Fan

**Affiliations:** ^1^Shaanxi Provincial Major Laboratory for Highway Bridge & Tunnel, Chang'an University, Xi'an 710064, China; ^2^School of Highway, Chang'an University, Xi'an 710064, China

## Abstract

In the past few decades, as a new tool for analysis of the tough geotechnical problems, artificial neural networks (ANNs) have been successfully applied to address a number of engineering problems, including deformation due to tunnelling in various types of rock mass. Unlike the classical regression methods in which a certain form for the approximation function must be presumed, ANNs do not require the complex constitutive models. Additionally, it is traced that the ANN prediction system is one of the most effective ways to predict the rock mass deformation. Furthermore, it could be envisaged that ANNs would be more feasible for the dynamic prediction of displacements in tunnelling in the future, especially if ANN models are combined with other research methods. In this paper, we summarized the state-of-the-art and future research challenges of ANNs on the tunnel deformation prediction. And the application cases as well as the improvement of ANN models were also presented. The presented ANN models can serve as a benchmark for effective prediction of the tunnel deformation with characters of nonlinearity, high parallelism, fault tolerance, learning, and generalization capability.

## 1. Introduction

**Figure 1:**
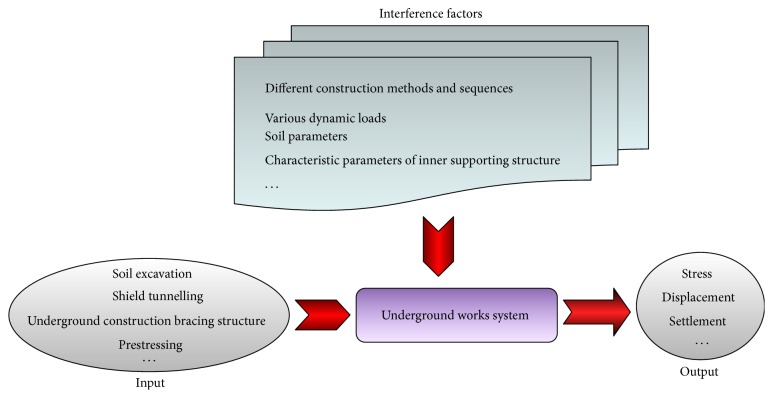
Underground works system frame.

Deformation prediction of the rock masses is one of the major subjects in determining the stability of the underground excavation projects. Recently, the tunnel construction is experiencing a very rapid growth in the complex geological formations and especially in urban areas where the low construction depth and the external loading from the buildings increase risk conditions [1]. When such conditions are not recognized prior to excavation of the tunnel, however, construction delays and increase of budge might occur. Therefore, reliable prediction of the soil deformation around the tunnel is crucial for preventing project setbacks [2]. Over a long period of time, most research efforts have focused on the regularities and mechanism of ground surface settlements and rock masses deformation, based on the accumulated experience and the in situ test data gathered from previous projects, which can reveal the stability of the tunnel. More specifically, the excessive deformation and structural failure can be significantly predicted by the dynamic information collection and monitoring in tunnelling. Then, according to the feedback information, the proper remedial measures can be employed in time [3]. Despite improvements made in the theoretical assessment of the tunnel deformation and the experiences gained from the monitoring data with different construction methods, there is still absence of reliable and targeted method of prediction available [4]. The empirical and analytical approaches cannot be appropriated for all geological situations and as they predict the deformation using only a limited number of geomechanical parameters and applying simplification, they cannot yield realistic outcomes [2]. Generally, to some extent, the engineering mechanics behavior of tunnel rock masses, consisting of the deformation and failure mechanism, is neither clarified nor readily predicted, by designers and engineers, due to the uncertainties in the geotechnical environments, the heterogeneity of the rock mass, and the deficiencies in the rock mass support interaction prior to construction, as shown in Figure 1.

Artificial neural networks (ANNs) commence as a new tool for analysis of the fuzzy geotechnical problems. The attractiveness of ANNs comes from the information processing characteristics of the system, such as nonlinearity, high parallelism, fault tolerance, learning, and generalization capability [5]. This technique allows generalizing from a training pattern, presented initially, to the solution of the problem. Once the network has been trained with a sufficient number of sample data sets a new input having a relatively similar pattern will be effectively predicted on the basis of the previous learning pattern [6]. Since the early 1990s, ANNs have been proposed as a way to address almost every problem in engineering [7, 8]. The literature reveals that ANNs have extensively been used to solve geotechnical problems such as modelling TBM performance [9], rock failure criteria [10], prediction of stability of underground openings [11], prediction of ground surface settlements due to tunnelling [12, 13], identifying probable failure modes for underground openings [14], prediction of tunnel support stability [15], tunnelling performance prediction [16], and prediction of tunnel rock masses displacement [17]. More specifically, Ghaboussi and Sidatra [18] first developed the constitutive model for geotechnical materials by using ANN. Considering the influence of the stress history and soil particle size, Ellis et al. [19] investigated the stress-strain relations for the sandy soils by employing the back-propagation (BP) networks. To improve the prediction accuracy, Shi et al. [20] applied a back-propagation neural network (NN) to predict the settlement induced by tunnelling or deep excavation. Hu et al. [21] studied the stability of the rock masses using the optimized MBP neural network. Kim et al. [22] investigated the ground surface deformation due to tunnelling using ANNs. Considering the monitoring of wall deflections of previous excavations stages and some geometrical characteristics, Jan et al. [23] developed a prediction model for further predicting the diaphragm wall deflection based on a neural network, which is capable of avoiding the trouble of assessing the soil parameters. Benardos and Kaliampakos [24] presented an attempt to model the advance rate of tunnelling with respect to the geological and geotechnical site conditions, which was implemented through the use of an ANN. Based on the influence of network training parameters on the quality of results, Santos Jr. and Celestino [25] analyzed the settlement over shotcrete-supported tunnels using the adjusted network, which was a good tool for predicting settlement above new tunnels to be excavated in similar conditions. For the sensitivity analysis, Shin et al. [26] proposed an index system for assessing the hazard level of collapse at a tunnel face employing a neural network based technique. With the aid of the ANNs, Armaghani et al. [27] successfully predicted the shear strength of rock. Additionally, Ahmadizar et al. [28] developed a new evolutionary-based algorithm to simultaneously evolve the topology and the connection weights of ANNs by means of a new combination of grammatical evolution (GE) and genetic algorithm (GA). Accordingly, unlike the classical regression methods in which a certain form for the approximation function must be presumed, ANNs do not require the complex constitutive models. This paper reviews the main developments in the field of tunnel deformation prediction system based on ANNs, along with their implications, key findings, and future research challenges. And the presented ANN frame models can serve as a benchmark for effective prediction of the tunnel deformation.

## 2. Artificial Neural Network Background

### 2.1. Basic Concept of ANN

**Figure 2:**
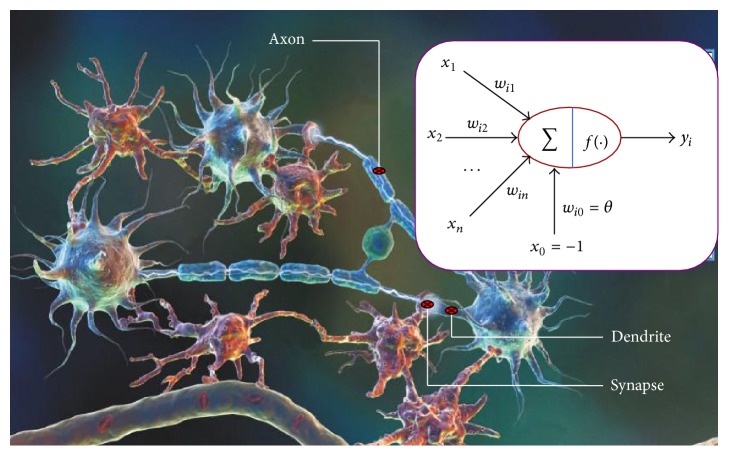
Biological neural networks.

ANNs are complex mathematical models inspired from biological neurons and are in fact the emulation of biological neural networks which are widely used in modelling of nonlinear systems and system identification, as shown in Figure 2. Generally, its powerful function of information processing depends on four factors, which are input-output properties (activation characteristics) of network units (neurons), network topologies (connection modes of neurons), connection weights (synaptic strength), and neuron thresholds (special connection weights). An ANN generally consists of an input layer, hidden layers, and an output layer. Every layer includes many neurons and the numbers of layers and neurons in each layer are determined by the user with respect to the scale of the problem. All neurons in the structure have interconnections between each other similar to biological nervous system [29, 30]. The ANN transfers the latent knowledge or laws in the related inputs to the network's site by processing the inputs and in fact it includes general laws based on the calculations performed on the numerical inputs or examples [31, 32].

### 2.2. ANN Architecture and Classification

#### 2.2.1. ANN Architecture

**Figure 3:**
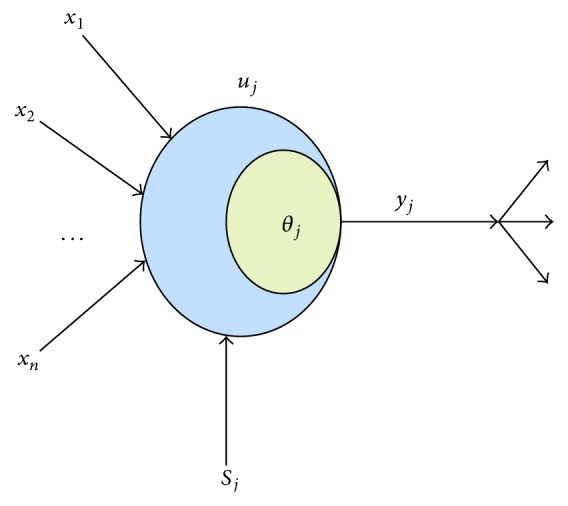
Artificial neuron architecture.

Traced back to 1943, the biological neurons had been actually lively presented by psychologist McCulloch [33] and mathematician Pitts. In order to simulate the functions of the biological neural network, the ANN model was established unprecedentedly [34], as shown in Figure 3.

The artificial neuron presented in Figure 3 is generally referred to as neuron or node. Where *u*_*j*_ is the internal state of the neuron, *θ*_*j*_ is the threshold, *x*_*i*_ is the input signal, *S*_*j*_ is the external input signal, *ω*_*ji*_ is the connection weights from *u*_*i*_ to *u*_*j*_, and *y*_*i*_ is the output signal. And the ANN model is established through many artificial neurons, which are connected by weights according to the connection mode.

#### 2.2.2. ANN Classification and Characteristics

In recent years, the number of ANN models proposed is far more than 60, which encompass BP, RBF, SVM, Hopfield, SOM, and so on. Apparently, these models have different characteristics and application areas [35, 36], as detailed in Table 1.

### 2.3. Modelling Using Back-Propagation (BP) Neural Networks

Currently, among various ANN models, radial basis function (RBF) model and back-propagation (BP) networks have gained wide application in tunnel deformation prediction.

#### 2.3.1. Radial Basis Function (RBF) Model

RBF method, as an interpolation technique in higher space dimensions, is a high-efficiency feedforward neural network, which has many advantages such as strong approximation property, better global optimization performance, simple structure, and fast training speed in comparison with other feedforward networks. Meanwhile, the RBF neural network has also been widely applied in the fields of pattern recognition and nonlinear function approximation.

#### 2.3.2. Back-Propagation (BP) Neural Networks

**Figure 4:**
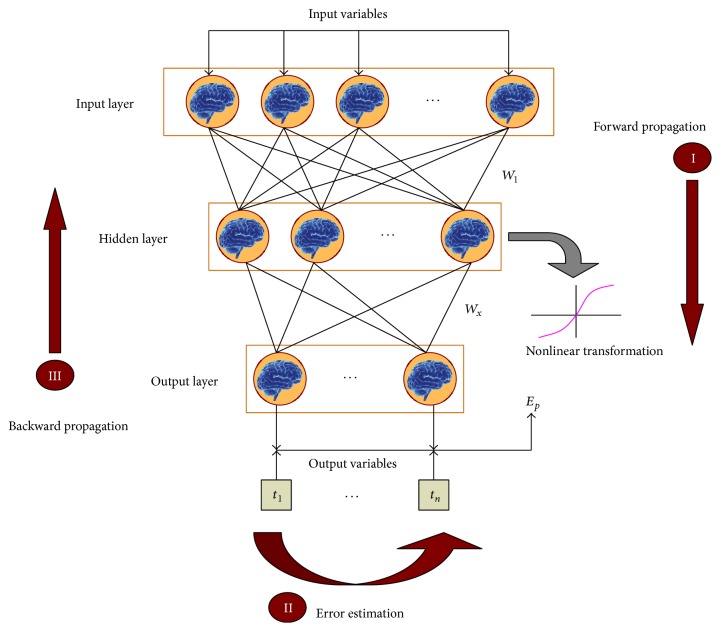
Typical structure of a BP network.

Typically, among the much different prediction models, BP neural network is the most popular used as a prediction method in tunnel projects for its nonlinear mapping approach capability, robustness, and easy realization, which has been used to correct the connection weights of network layers from the posterior layer to the anterior layer utilizing the difference between real output and desired output. BP network is a typical multilayer network [33], which consists of three layers: input layer, hidden layer, and output layer [20], depicted in Figure 4. More specifically, in an ANN model, each neuron receives signals from the environment or other neurons. The signals are summed in the neuron and are subsequently transmitted to the connected neurons.

BP network was firstly proposed by Werbos [38], which is based on searching an error surface (error as a function of ANN weights) using the gradient descent algorithm for points with minimum error, and more attentions have been attracted in the middle of the 1980s. Since the perceptron [39] (an initial learning algorithm of ANN) can only address simple issues in pattern classification, the BP network within a multilayer ANN is capable of approximating any nonlinear function. More importantly, it is determined that Hecht-Nielsen [40] has proved that one hidden layer of BP neurons suffices to model any solution surface of practical interest. Increase in number of neurons in hidden layer has positive optimizing in the performance of BP network and a hidden layer of more than 10 neurons does not exert a significant effect on optimizing the performance of the neural network.

**Figure 5:**
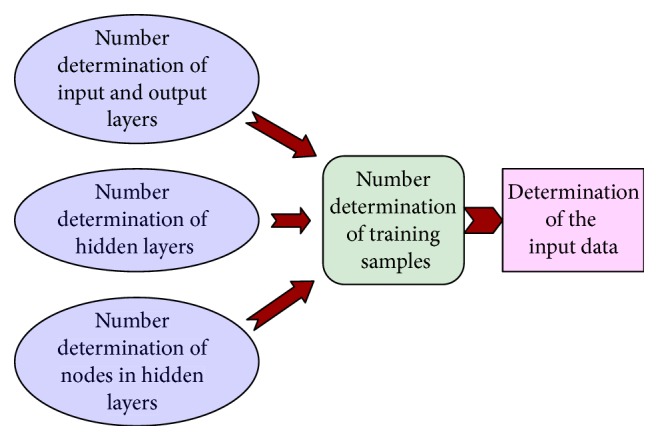
BP network design frame.

Designing a BP network architecture includes determining the number of input and output variables (i.e., neurons in input and output layers) and selecting the number of hidden layers and neurons in each hidden layer, as shown in Figure 5. The number of hidden layers and number of neurons in each hidden layer in a BP network may affect the training efficiency and the precision of prediction. The number of neurons in the input and output layers corresponds to the expected input and output variables of the problem. Output variables are the expected answers to the problem, and the input variables are factors that affect the outcomes [20]. And then the samples, used for the learning and memory of the system, in BP network should be determined. Furthermore, the number of the samples should meet the learning requirements, which is determined by the specific research questions. With the increase of influence factors and outputs, the training samples also correspondingly increase.

**Figure 6:**
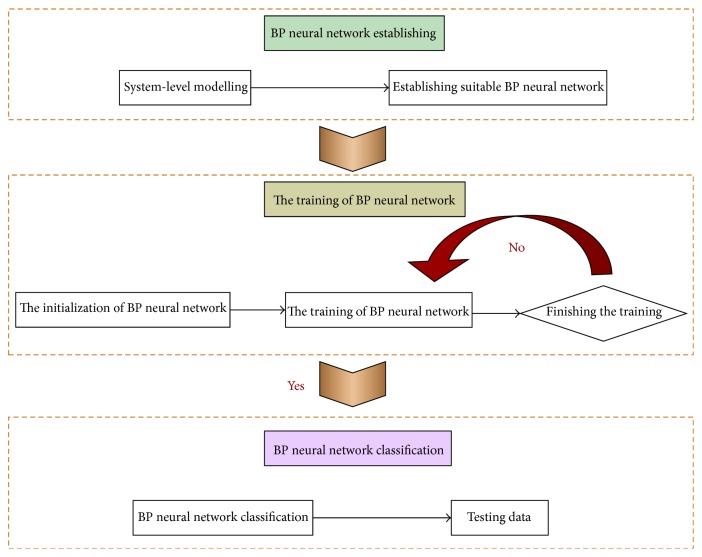
Procedure of the design of BP neural network.

The input data need to be normalized before the training of samples, and then the data are reflected to initial ranges after the training of samples. Based on the* postreg* function of MATLAB, Yang [41] performed the linear regression analysis of the training results of the built network and obtained the optimal normalization intervals. Besides, specific functions in the network layers are determined by specific research questions. Based on input samples, the learning and debugging process of BP neural network is developed. Considering the significant influence of the initial position of training model on the convergence of BP neural network, we can avoid the local minimizer during the convergence process by changing the number of the network neurons or adding a random number to each connection weight. The procedure of the design of BP neural network [42] is given in Figure 6.

## 3. Tunnel Deformation Prediction System

### 3.1. Basic Principles in ANN Prediction System

Currently, the underground engineering problems based on ANNs are mainly classified as two types: one is the nonlinear relationship of parameters, which is established by the given data; the other is the prediction system for the latter period based on the experience gained and the in situ test data gathered from past projects.

**Figure 7:**
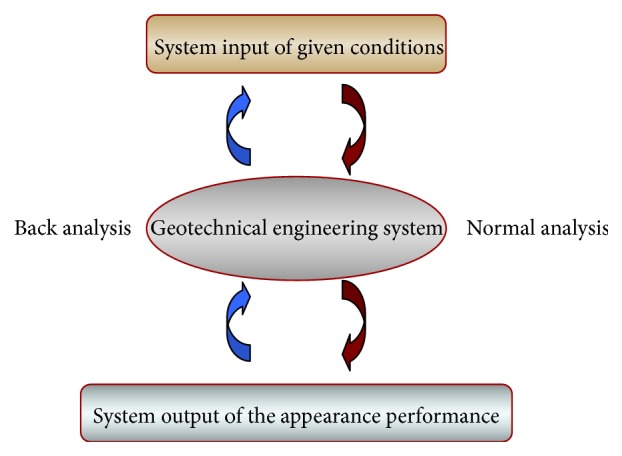
Geotechnical engineering system [37].

For the prediction system, the geotechnical engineering is certainly a complex giant system, as shown in Figure 7. Specifically, in order to know the deformation mechanism of the rock masses and ensure the construction safety, we can predict the displacement and deformation in front of working face through various known conditions; on the other hand, we can perform the back analysis of construction conditions through the given displacement and deformation.

### 3.2. Deformation Factors in Tunnelling

**Figure 8:**
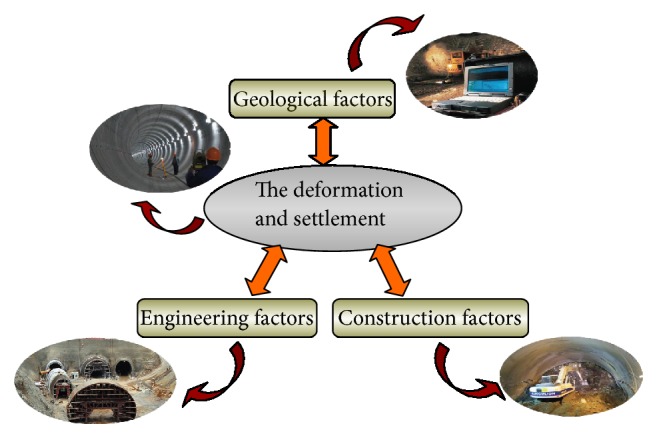
Interaction of deformation factors.

Generally, excessive deformations in tunnels are induced by the rock mass disturbance. For mountain tunnels, loess tunnels, or urban-road shield tunnels, the ground surface settlement and the deformation induced by tunnelling are mainly composed of three factors, as shown in Figure 8, which are geological factors, engineering factors, and construction factors. As detailed, the geological factors mainly consist of rock mass grade, Poisson's ratio of rock and soil masses, angle of internal friction, joint development in rock, soil moisture content, and so forth. Engineering factors mainly consist of tunnel depth, design dimension, span-to-depth ratio, support stiffness, and so forth, whilst construction factors mainly consist of construction approach, shoring time, boring parameters, and so forth. Moreover, there is a significant interaction among the various factors; for instance, the deformation of the rock masses will decrease with the big stiffness of support system when the stability of geology is quite insufficient, while the deformation will increase with unsuitable construction approach and shoring time.

Considering the different geological conditions, scale of projects, and construction approaches in the research of practical problems, we usually regard the specific and dominant influencing factors as input variables in BP networks. Meanwhile, the deformation to be predicted is taken as the target variable. In order to select the effective input variables, we should follow the two basic principles: (1) independent variables selected should be closely related to the target variables; (2) there should be not significant linear relationship among the independent variables.

### 3.3. ANN Prediction Model for Tunnel Deformation

For the given time series {*X*(*t*), *t* = 1,2,…, *n*}, the tunnel deformation prediction model can be given by the following expression [43]:

(1) $$\begin{array}{c} X\left(\;t\;\right)=\varphi \left[\;X\left(\;t-1\;\right),\ldots ,X\left(\;t-p\;\right)\;\right], \end{array}$$

where *φ*[·] is the nonlinear function and *p* is the model function. Actually, the major task of the prediction model is to obtain the suitable nonlinear function. Based on the in situ test data gathered from previous projects, *φ*[·] can be inversed rapidly and accurately with the reasonable structural parameters of ANNs.

**Figure 9:**
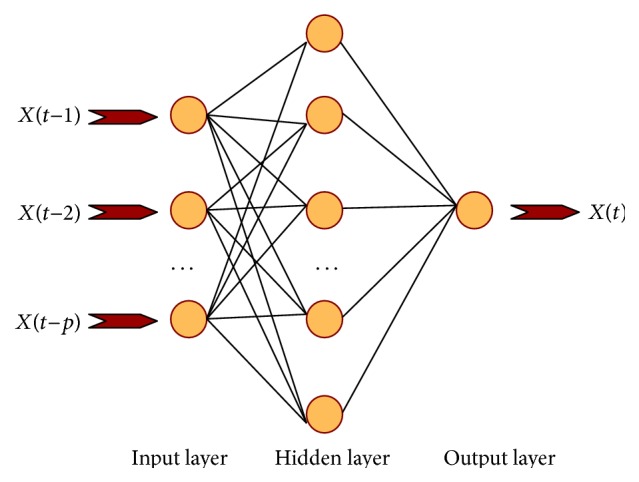
Multiple-input single-output BP network model.

In order to predict the rock mass deformation in tunnelling, a multiple-input single-output BP network model is established, as shown in Figure 9. And the *n* − *p* samples are constructed from the given time series {*X*(*t*), *t* = 1,2,…, *n*}, as shown in Table 2.

Afterwards, samples learning and training based on BP network are conducted. The stable network structure, connection weights, and neuron thresholds can be obtained after learning and training. Then the tunnel deformation prediction model based on BP network can be established. When the variables of samples to be predicted are input to the BP network, the output variables of samples can be obtained correspondingly.

Because of the complex relationships among these factors which have influences on the soil deformation, it is very important to select the influence factors when the model is established. In the prediction model, the output variables are the solutions to be desired; meanwhile, the input variables are the influence factors of the output variables. The number of neurons in the input layer should be designed according to the requirements of the output layer. The size and range of the original space are determined by the input layer of the network, and the number of neurons in the input layer should be matched with the field monitoring data. The successful application of the networks depends on the full knowledge of problems to be solved. Therefore, what the problem to be solved by using the neural network must be clear first; then, based on the field monitoring data, several issues need to be addressed: input variables which determine the problem, output variables which are the solutions to the problem, value range of variables, and corresponding results. After the problem is determined, the number of neurons in the input layer and output layer is determined accordingly.

With regard to the number of neurons in the hidden layer of BP network, it is important to note that the number is obtained mainly through the accumulated experience and the in situ test data gathered from past projects without an exact analytical expression. And the determination of the number of neurons should be in conformity with two basic principles of less iterated number and strong fault tolerance. Additionally, for the general BP network, the number of neurons in the hidden layer can be obtained as follows [44, 45]:

(2) k<∑i=1nCn1i,n1=n+m+c,n1≥log2⁡n,

where *n*_1_ is the number of neurons in the hidden layer, *n* is the number of neurons in the input layer, *m* is the number of neurons in the output layer, *k* is the number of samples, *C* is a constant in the range of 1–10, and $C\left[\begin{array}{c} n_{1}\\ i \end{array}\right]$ (*i* < *n*_1_) is the combination symbol. If *i* > *n*_1_, $C\left[\begin{array}{c} n_{1}\\ i \end{array}\right]=0$.

### 3.4. Prediction of Ground Surface Settlement due to Shield Tunnelling

**Figure 10:**
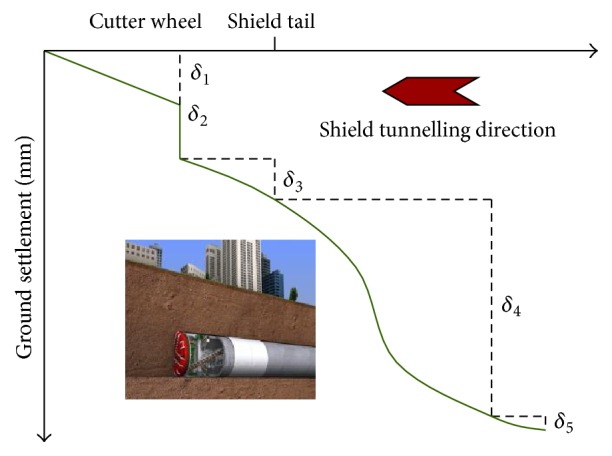
Ground settlements in different construction stages [46].

Since the first shield tunnel was completed in London 170 years ago, shield tunnelling is greatly popular with its flexibility, cost-effectiveness, and little impact on ground traffic and surface structures [47]. One of the key factors in shield tunnelling is the prediction of ground surface settlement since very often construction is undertaken in densely populated urban areas with heavy traffic and public utilities, such as storm drain, sewer, steam, water, gas pipes, and electrical and telephone ducts [48]. And the ground settlements in different construction stages are shown in Figure 10.

Typically, for the aforementioned problems, Qu [49] has predicted the ground settlement during shield tunnelling using an ANN model with 9 neurons in the input layer, 2 neurons in the output layer, and 12 neurons in the hidden layer. Based on the actual ground settlements induced by shield tunnelling in Shanghai, Guangzhou, Nanjing, and so forth, the training samples are selected to finish the neural network training. Shanghai Metro Line 2 (Longdong Road-Zhongyang Park) is located in soft soil layers of clay, sandy silt, silty clay, and muddy clay. The outer diameter of the soil pressure balanced shield is 6340 mm. Guangzhou Metro Line 2 (Yuexiu Park-Sanyuanli) mainly consists of two regional tunnels, which are located in the soil layers of weak weathered rock, deeply weathered rock, and residual soil. The outer diameter of the soil pressure balance boring shield machine is 6250 mm. The thick soft soil layer is distributed at Nanjing Metro Line 1 (Xuanwu Gate-Xinmofan Road), which is mainly composed of the uneven soils including low plasticity silty clay and silty sand. The tunnel is located in the soil layers of silty clay and fine sand with the complex engineering geology. The outer diameter of the shield machine is 6400 mm.

**Figure 11:**
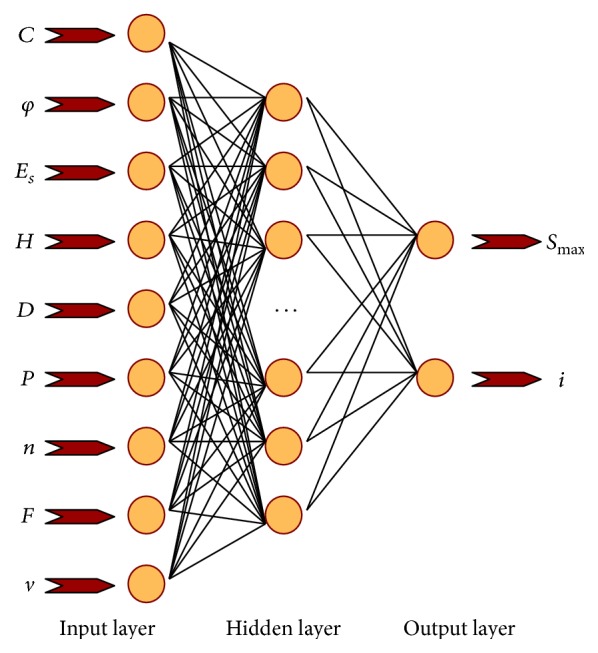
Prediction model of shield tunnel ground deformation.

The training samples selected are given in Table 3. More specifically, the input variables consist of cohesion (*C*), angle of internal friction (*φ*), compressive modulus of soil (*E*_*s*_), earth covering thickness (*H*), diameter of TBM (*D*), grouting pressure (*P*), grouting filling ration (*N*), shield jacking force (*F*), and shield tunnelling rate (*v*). Meanwhile, the output variables are composed of the maximum surface settlement (*S*_max⁡_) and width coefficient of settling tank (*i*). Accordingly, the prediction model based on ANN can be established, as shown in Figure 11.

**Figure 12:**
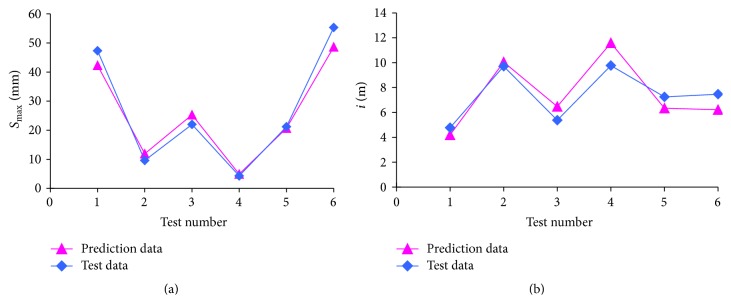
Prediction results of training samples.

Then, the samples learning and training are conducted using neural network toolbox in MATLAB, which can be used to establish the nonlinear relations between inputs and output parameters. And the prediction results, as shown in Figure 12, are perfect, significantly verifying the effectiveness of this model.

From the prediction results, as shown in Table 4, it can be seen that the maximal absolute error of *S*_max⁡_ is 6.70 mm, the maximal relative error of *S*_max⁡_ is 24%, the maximal absolute error of *i* is 1.82 m, and the maximal relative error of *i* is 20%. Furthermore, the mean absolute errors of *S*_max⁡_ and *i* are 3.1 mm and 1.00 m, respectively, and the mean relative errors of *S*_max⁡_ and *i* are 13.0% and 14.2%, respectively. Generally speaking, it can be concluded that this prediction model based on ANN can obtain high-precision results.

**Figure 13:**
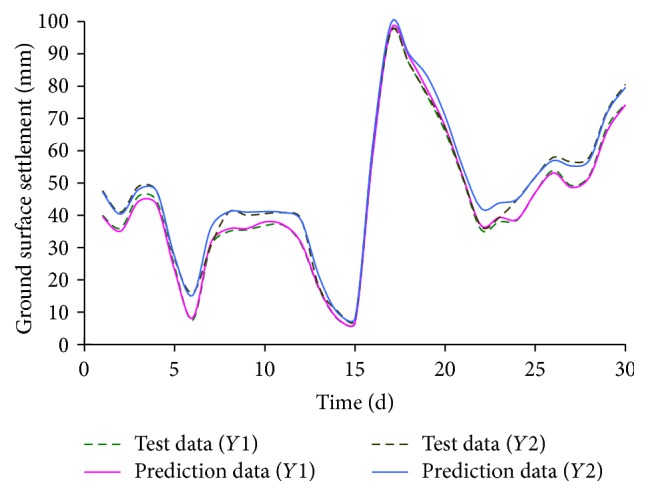
Simulative results of training samples.

**Figure 14:**
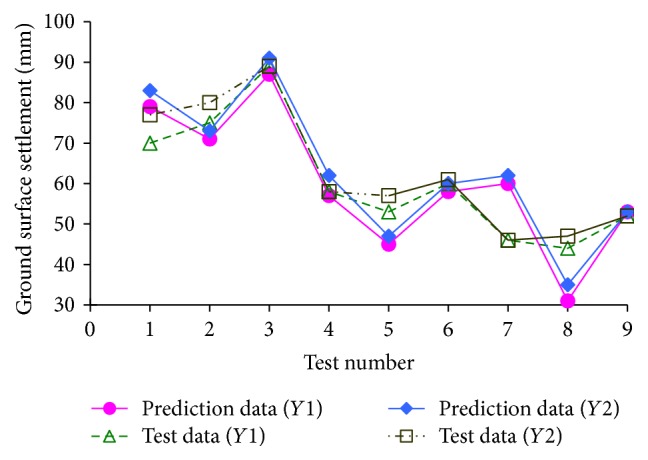
Simulative results of test samples.

Additionally, Sun and Yuan [46] studied the mechanism of ground movement and ground surface settlement induced by soil disturbance in the shield tunnel construction of the city metro-subway by using artificial intelligence ANN technology. The established ANN model can accurately predict the ground surface settlement in front of the working face of 5 m. In order to reduce the soil disturbance and ground surface settlement, the shield tunnelling parameters should be corrected timely when prediction results are received or the alarm occurs in shield tunnelling. Simulative results of training and test samples are shown in Figures 13 and 14. From the simulative results, it can be seen that the maximal absolute error and relative error of prediction data are 16 mm and 34%, respectively. Meanwhile, the mean absolute errors of output parameters (*Y*1 and *Y*2) are 5.56 mm and 6.01 mm, respectively. Correspondingly, the mean relative errors of *Y*1 and *Y*2 are 9.2% and 9.5%, respectively. Note that *Y*1 is the stratum loss in front of the working face of 5 m; *Y*2 is the ground surface settlement in front of the working face of 5 m. The simulative results indicate that the established ANN model can provide high prediction accuracy in ground surface settlement induced by rock mass excavation.

**Figure 15:**
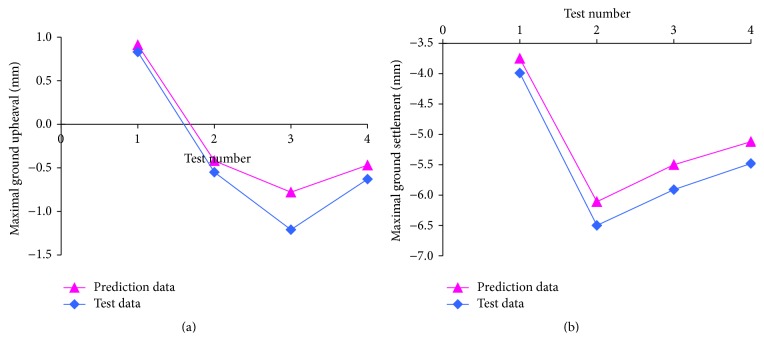
The maximal ground upheaval and settlement change in the targeted area with tunnelling of 10 m.

Moreover, concerning the shield tunnelling construction and measurement development in time sequence, Wang et al. [50] established an ANN model to predict the ground deformation with the conception of the receding optimization, which was realized by partial least squares networks. The variations and total settlements can be significantly predicted according to the monitoring data from previous sections. And the dynamic application of this model in two overlapped tunnels of the Shanghai metro projects was successfully realized. The soil pressure balanced shield machine of mudding type was used in the Shanghai metro engineering. Considering that the unilateral drive system was employed in the station structure of the metro transit project, two separated tunnels gradually came close to each other and finally overlapped in vertical profile in the metro station. On the basis of the field data of the ground surface settlement during shield tunnelling, a total of 23 samples were determined, and 19 samples were selected for network training, and the other 4 samples were selected for network testing. As shown in Figure 15, for training results, the error was controlled within −0.80~1.36 mm, and, for verification results, the error was controlled within −0.36~−0.14 mm, which indicated that the ground surface displacements could be significantly predicted by the ANN model.

From the previous section, it can be seen that the prediction of ground surface settlement due to shield tunnelling using the ANN models is effective concerning the combined influence of factors, for example, geological environment, physical parameters of TBM, and construction craft.

### 3.5. Deformation Prediction in NATM Tunnelling

According to NATM, the monitoring and measurement are crucial for safety evaluation in NATM tunnelling. Typically, the tunnel deformation monitoring during excavation is relatively an important item, especially tunnelling in harsh geological areas.

**Figure 16:**
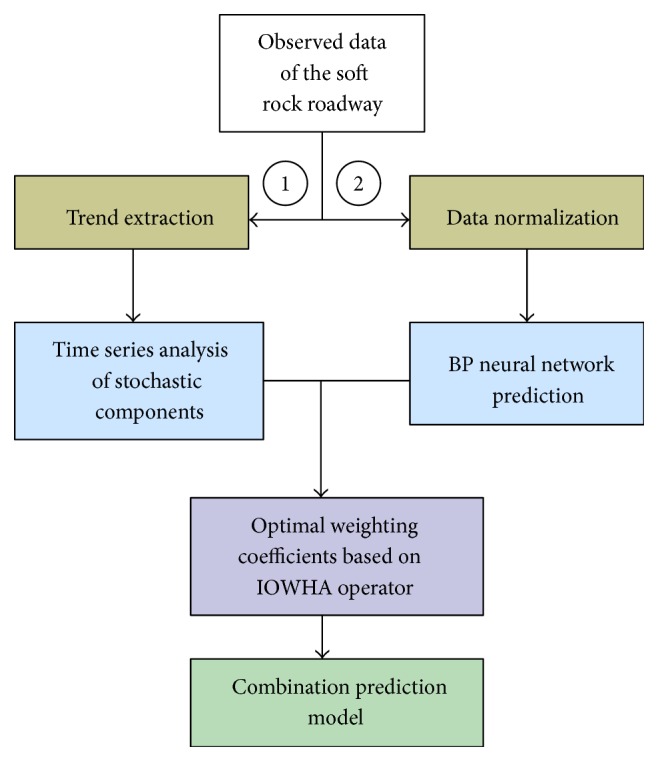
Procedure of the design of combination prediction model [52].

Recent years have seen an increased interest in the prediction of tunnel performance, notably the rock mass deformation. For the prediction of rock masses deformation in front of the working face according to the given geological and construction conditions, a substantial research effort has been undertaken. Based on results of FEM simulation, Chang et al. [51] predicted the stresses and deformations of rock masses during tunnelling using an ANN method. Furthermore, the algorithm of the BP network technique was improved appropriately and several new improving methods were also developed, which could obtain more accurate and more feasible results for practical applications. Considering that traditional deformation analysis methods are difficult in overcoming their defects, such as singleness and low accuracy, Xiong and Meng [52] established a combination prediction model employing the BP neural networks integrated with the time series analysis, which was used to predict the deformation of soft rock roadways. Based on the results from the single method, the weight of each method was calculated according to IOWHA operator in the combination prediction model. And the procedure of the design of combination prediction model is shown in Figure 16. Finally, the deformation rate of rock masses was obtained, which could efficiently judge the stability of rock masses. Additionally, this new combined prediction method has much more advantages of timing analysis than ANN methods, and the prediction precision of the combination prediction model is improved obviously.

**Figure 17:**
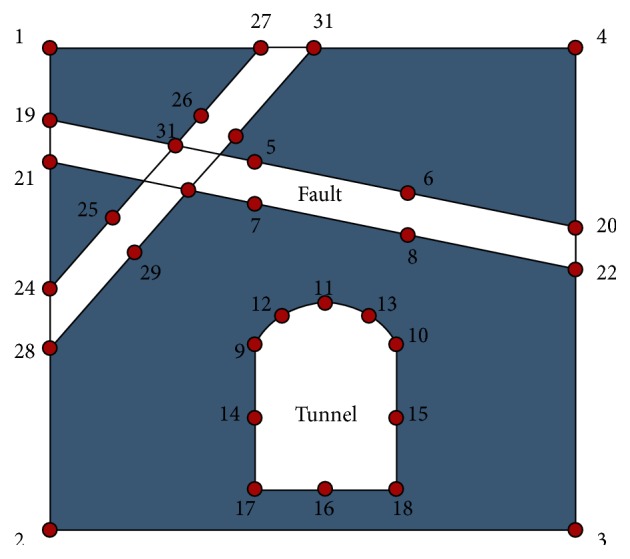
Key points for displacement field of underground tunnels [53].

On the basis of predecessors' research results on the tunnel deformation, Jian [53] analyzed the characters of deformation and stability of underground caverns with nonfault, single fault, and double faults using the BP neural network. And the locations of key points, as shown in Figure 17, were selected from a large number of numerical investigations. Finally, the deformation prediction model of key points based on BP neural networks was established, which can predict distortion of rock masses when the deformation modulus, Poisson's ratio, cohesion, internal friction angle, chamber depth, cross-hole, high-span ratio, and coefficient of lateral pressure were arbitrary. Through the domain decomposition method, the displacements in different directions of each key point were trained in a small prediction model with 9 neurons in the input layer and 1 neuron in the output layer. Generally, this prediction model based on BP neural network is practicable with the good network performance and fast convergence rate. The network extension of this prediction model, however, is far from perfect, which needs to be further optimized.

The neural network for double faults model, which had the highest prediction accuracy, was selected after 64 neural networks for *X* and *Y* displacement prediction of 32 key points were tested. And the prediction errors of neural networks for *Y* displacements of key points 11, 14, and 15 are presented in Table 5.

### 3.6. Displacement Back Analysis Based on ANNs

The normal analysis (prediction of the deformation induced by the tunnelling through the given mechanics parameters of rock masses) and the back analysis (prediction of the mechanics parameters of rock masses through the given deformation) are closely interrelated and inseparable from each other, especially when the mechanics parameters of rock masses are difficult to be obtained and the number of that is limited in the practical projects. Thus, the prediction of the mechanics parameters of rock masses through the in situ test data is of great significance [54]. Suwansawat and Einstein [55] reported how ANNs were used to determine correlations between TBM operational parameters, ground mass characteristics, and surface movements. They emphasized that one of the greatest difficulties in the analyses was obtaining all the parameters that may be related to the instrumentation. Aiming at practice in site, Hao and Liu [56] proposed a new back analysis method of mechanics parameters of rock masses by calculus of differences and ANNs. Training was performed by ANNs, and the training parameters were optimized. By the given displacements, mechanics parameters of surrounding rock were significantly predicted and contrasted. Moreover, the precision of calculated results was satisfied, which confirmed correctness and reliability of the back analysis method. Zhou et al. [57] conducted the back analysis by means of the BP neural network algorithm on the mechanics parameters of rock masses in a highway tunnel construction project by NATM. The weight value matrices and tolerance vectors in accordance with input neurons and outputs were obtained by training a set of specimens, which came from an FEM analysis for the tunnel. And the analogy results of the BP network produced a good agreement with the FEM calculations. Concerning the realization of the defects in the general BP neural network, Gao and Xie [58] proposed an approach based on improved BP neural network for displacement back analysis of soft and weak rocks. This BP neural network was improved in two ways, neural structure and algorithm, by using increased feedback in output layer, determination of the number of neurons in hidden layer according to dichotomy theory, improved inertia revision, varying step-size algorithm, and improved error function. Consequently, the corresponding error between monitoring data and training results was controlled within 4%.

**Figure 18:**
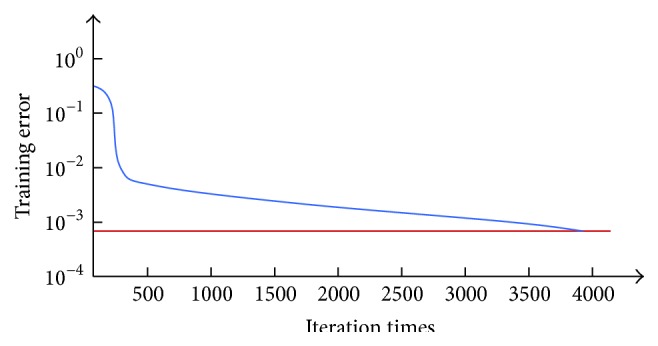
Training error curve in MATLAB.

Typically, in order to address the problems of the model complex and slow speed in problem solving for all conventional displacement back analyses, Yun et al. [59] established an ANN model that could be used for back analysis of mechanical parameters of tunnel rock masses using the program of BP neural network compiled by the M language of MATLAB. Considering the disadvantages of slow-footed convergence of the traditional BP neural network, optimization arithmetic and normalization methods were used to accelerate the network training rate. Based on the monitoring data (vault settlement and horizontal convergence), the samples were input in the BP neural network for training. And the mean square error of the output was tending towards stability after 3888 iterations, as can be seen in Figure 18. Specifically, the analyses results are given in Table 5.

From Table 6, the mean corresponding errors between the monitoring data (*E*, *μ*, *φ*, *c*) and training results (*E*, *μ*, *φ*, *c*) can be obtained with a high precision, which are 7.27%, 1.37%, 2.94%, and 9.17%, respectively.

The model established is simple which readily addresses the problem and is more likely to get accurate solutions. What is more, it can be commonly used in underground engineering for displacement back analysis. The BP algorithm is probably used to inverse the mechanical parameters of tunnel surrounding rock. The application of back analysis method is indispensable in the stability analysis of the tunnel rock masses and in informational design.

## 4. Comparison and Improvement of ANN Models

### 4.1. Comparison of ANN Model with Other Systematic Models

Unlike the classical calculation methods in which a certain form for the approximation function must be presumed, ANN models for tunnel rock masses present better flexibility, stronger nonlinearity, and lower values of prediction error; details are as follows:

1. The qualitative descriptions of rock mass properties, such as rock mass grade, weathering degree, and influence degree of geological structure, can be taken as the input variables. Moreover, there is no limitation of the number of the input variables.
2. Parameters processing and prediction accuracy can be modified manually and the data can be incomplete and inaccurate.
3. It is effective to address problems without certain solution methods.

On the subject of the deformation prediction in tunnelling projects, except for the ANN approach, extensive numerical investigations, which include finite element method, boundary element method, and semianalytical method, have been performed. Considering the large amount of calculation and the inaccuracy of modelling, however, numerical methods have many limitations and defects, especially for the deformation prediction during the real tunnel excavation. Typically, based on the experience gained from past projects, Zhou [60] developed a shield tunnelling expert system (STES), which was applied to investigate the influence of shield tunnelling on the surrounding environment. This expert system, however, has large difficulty in gathering massive monitoring data and experience from engineers. The grey system theory, additionally, has also been applied in the deformation prediction during tunnelling. The grey exponential law of the time series should be satisfied in the grey modelling; however, the accumulative generation for many monitoring data generally does not follow the grey exponential law, which directly causes large prediction error. In order to predict the deformation of the support construction-underground continuous wall in the process of pit construction, Zhao et al. [61] systematically introduced an ANN prediction method of multistep tumble skill based on time window and discussed a series of key technique problems, such as input and output layer design, the proper number of hidden layer neurons, and time-step of prediction. Obviously, it can be seen that ANN prediction models are superior to other methods, such as regression analysis and grey system theory. In view that most of the models focused on the maximum ground surface settlement and did not involve dynamic and real-time predictions, Wang et al. [62] investigated how tunnelling-induced ground surface settlement developed using a smooth relevance vector machine with an ANN system. Unlike conventional methods that require additional efforts to determine relevant model parameters, the proposed method can optimize the parameters in the training process. In addition, it can be concluded that the prediction capacity of ANN is better than the empirical models developed previously. Nonetheless, the ANN prediction models are not always employed universally. Yao et al. [17] developed the SVM (support vector machines) model and ANN model to predict tunnel rock mass displacement. By comparison, it can be found that the regression level of SVM model is better than that of ANN model. Based on the integration between wavelet theory and ANN, or wavelet neural network (WNN), Chen et al. [63] proposed a local linear wavelet neural network (LLWNN). The difference of the network with conventional WNN is that the connection weights between the hidden layer and output layer of conventional WNN are replaced by a local linear model. And the simulation results show the feasibility and effectiveness of that proposed method. Practically, the ANN method coupled with other prediction methods has provided new insights into the improvement of the accuracy and efficiency in the calculation system, save the manual time and labor resources.

### 4.2. Limitations and Development of ANN Methods

Although the development of BP neural networks for modelling and prediction of performance of mechanical excavators, such as TBM, and circular saw machines, has been well accepted through the scientific community, they still have some limitations including the slow rate of learning, “black box” nature, greater computational burden, proneness to overfitting, and entrapment in local minima [64]. Because of the incomplete of influence factors in ANN models, the application of ANNs is limited. In construction projects, engineering geological parameters are selected according to the investigation experiences in different areas, which have significant difference from the actual parameters. It is well known that the data processing for the soil deformation prediction is conducted using the mathematical models established through the monitoring data; however, other factors play an important role as well. Further studies are needed to be carried out to make the model more perfect, which means more influence factors should be fully taken into consideration and the approach on collecting samples should be optimized. For the deformation prediction in geotechnical engineering, ANNs are employed to predict the deformation of next sites according to previous monitoring data. Neural network models are quite accurate for prediction of the soil deformation in a near time, while the prediction in a long time is relatively inaccurate. Therefore, further studies on the prediction theory and models need to be conducted.

In recent years, great improvements have been made in this area, and many powerful methods have been developed for the prediction of performance with high efficiency in tunnelling. For instance, Yan [65] proposed an expert system based on ANNs for selecting methods of tunnel construction by combining the expert system and BP neural network. Feng et al. [66] developed an evolutionary neural network method for displacement back analysis by combining the neural network and genetic algorithm, which could commendably manage the relationship between the rock mechanical parameters and deformation due to tunnelling. By the introduction of the present situation of back analysis and its intelligent study, Gao and Zheng [67] displayed that some problems existed in the back analysis and proposed an integrated intelligent method combined with the ANNs. It can be concluded that artificial intelligent techniques are developing towards the intelligent integration. Additionally, based on the responding composition model, the displacement of tunnel rock masses was predicted by Grey-ENN model described in [68], which combined the advantages of GM (1, 1) and ANN model. Importantly, GM (1, 1) can predict the trend of increasing sequence, and ANN model can reduce error by self-adapting of network. And the literature [68] indicated that the results obtained were highly encouraging and satisfactory compared to the conventional ANN methods. These results provide insight into ways of improving the efficiency and generalization capability of ANN prediction models. Through the optimization of monitoring and analyzing for the prediction models, there is reason to believe that the intelligent control technology, consisting of artificial intelligence, expert system, fuzzy control algorithm, neural network, genetic algorithm, and so forth, would be probably employed for the development of intelligent integration control system in road tunnels, such as blasting vibration monitoring system [69], illumination intelligent control system [70], and prediction and early-warning of geological hazards.

## 5. Concluding Remarks

Due to the heterogeneity and nonlinearity of the rock mass, the prediction of the rock mass deformation in tunnelling is indispensable for optimization of the tunnel construction while simultaneously observing the safety requirements. For this purpose, ANN has been successfully applied in the deformation prediction and displacement back analysis, which seems to have good potential of time-saving and cost-effectiveness. The present paper reviews the state-of-the-art of the field of ANN technology in tunnel performance prediction. By the application case analysis and summary mentioned above, we can obtain the following conclusions and perspectives.

(1) ANNs have some extruding advantages, including self-learning ability, self-organized ability, high nonlinearity, good fault tolerance capability, and calculation inaccuracy. The application case analysis shows that the prediction of the rock mass deformation based on ANNs has a preferable applicability. In other words, the ANN prediction modelling is one of the most effective ways to predict the rock mass deformation, which is more practical for the dynamic prediction of displacements in tunnelling.

(2) Currently, the application of ANNs in the engineering field is well known to engineering sciences with the mature technology. And the neural network toolbox of the MATLAB software has been used for building the BP network code to establish the model. In this way, the relationship between the dependent variable of deformation and the independent variables of geomechanical parameters can be established. Obviously, it can be envisaged that ANNs would be more suitable for the analysis of engineering problems in the coming years, especially if ANN models are combined with other research methods.

(3) What is crucial in the BP network modelling for prediction of the rock mass deformation in tunnelling is finding the influence factors. These influence factors, as input variables, must be endowed with dynamic changes and be easy to obtain. Meanwhile, the monitoring data for network training must be sufficient, accurate, and scientific, which should be modified or rejected if necessary. The prediction ability, additionally, can be improved when the content of samples increases.

(4) Application of ANNs combined with many finite element software programs, consisting of ANSYS, ABAQUS, MIDAS/GTS, and so forth, can be readily used to pinpoint the sensitive factors which determine the deformation of rock masses in tunnelling. According to the previous finite element analysis, however, it is generally hypothesized that the rock mass is homogenous and continuous, which is quite different from actual field conditions. Therefore, the ingathering of training samples as well as precision discrimination of output variables should be considered carefully.

(5) In most cases, however, ANN prediction system has the limitation of time-lag effect. Based on the experience gained and the in situ test data gathered from previous projects, we can develop the ANN prediction system for the prediction of engineering problems in the later construction stages. Finally, taking our results into consideration, we reasonably believe that the future prediction system of the rock mass deformation based on ANNs would be more real-time, dynamic, and successive, which holds great promise for the construction safety.

## Figures and Tables

**Table 1:** Application of ANN models.

Attribute	Neural network				
	BP	RB	SVM	Hopfield	SOM
MATLAB function	Newff	New, Newrbe	Svmpredict	Newp	Newc
Application area	Approximation Prediction classification	Approximation prediction	Prediction classification	Classification	Classification

**Table 2:** Sample construction of BP network.

Sample	Input layer vector	Output layer vector
The first sample	*X*(1),…, *X*(*p* − 1), *X*(*p*)	*X*(*p* + 1)
⋮	⋮	⋮
The (*t* − *p*)th sample	*X*(*t* − *p*),…, *X*(*t* − 2), *X*(*t* − 1)	*X*(*t*)
⋮	⋮	⋮
The (*n* − *p*)th sample	*X*(*n* − *p*),…, *X*(*n* − 2), *X*(*n* − 1)	*X*(*n*)

**Table 3:** Training samples for ground settlement prediction [49].

Test number	*C/*kPa	*φ/*°	*E*_*s*_/MPa	*H/*m	*D/*m	*P*/MPa	*N/*%	*F*/MN	*v/*mm·min^−1^	*S*_max⁡_/mm	*i/*m
1	11.8	15.2	5.91	9.78	6.34	0.35	1.7	14	20	47.3	4.77
2	28.1	32.8	5.42	13.3	6.40	0.25	1.5	31.65	40	9.6	9.71
3	32.4	10.7	11.17	14.5	6.40	0.25	1.7	31.65	60	22.0	5.37
4	31.1	44.1	40.75	20	6.25	0.30	1.3	33	30	4.3	9.77
5	11.9	13.8	5.22	11.9	6.34	0.40	2.0	14	20	21.2	7.25
6	12.1	13.7	5.21	12	6.34	0.35	1.7	14	30	55.3	7.46

**Table 4:** Comparison between prediction results and in situ test data [49].

Test number	*S*_max⁡_/mm				*i*/m			
	Prediction data	Test data	Absolute error	Relative error	Prediction data	Test data	Absolute error	Relative error
1	42.3	47.3	−5	−0.11	4.19	4.77	−0.58	−0.12
2	11.9	9.6	2.3	0.24	10.05	9.71	0.34	0.04
3	25.3	22	3.3	0.15	6.47	5.37	1.1	0.2
4	4.9	4.3	0.6	0.14	11.59	9.77	1.82	0.19
5	20.7	21.2	−0.5	−0.02	6.34	7.25	−0.91	−0.13
6	48.6	55.3	−6.7	−0.12	6.21	7.46	−1.25	−0.17

**Table 5:** *Y* displacement of key points.

Test number	Point 11			Point 14			Point 15		
	Prediction data (mm)	Test data (mm)	Error (%)	Prediction data (mm)	Test data (mm)	Error (%)	Prediction data (mm)	Test data (mm)	Error (%)
1	−5.12	−4.46	14.8	−1.33	−1.76	24.43	−1.12	−0.78	43.59
2	−16.21	−11.71	38.43	−4.25	−5.43	21.73	−3.45	−3.23	6.81
3	1.93	2.22	13.06	−0.78	−0.60	30.00	−22.56	−27.23	17.15
4	63.38	59.16	7.13	−82.24	−90.55	9.18	−45.68	−40.88	11.74
5	0.75	0.57	31.58	−0.52	−0.23	126.09	−1.21	−1.16	4.31
6	−5.28	−5.77	8.49	−11.37	−9.38	21.22	−1.16	−1.06	9.43
7	−7.01	−6.79	3.24	−3.26	−2.14	52.34	−2.15	−1.79	20.11
8	−113.42	−107.30	5.70	−23.29	−26.49	12.08	−21.64	−26.50	18.34
9	−72.22	−64.17	12.54	−13.04	−13.20	1.21	−14.06	−13.17	6.76
10	−0.94	−0.67	40.30	−1.61	−1.75	8.00	−0.44	−0.25	76.00

**Table 6:** Comparison between monitoring data and training results [59].

Sample	Monitoring data				Training results			
	*E*/GPa	*μ*	*φ*/(°)	*c*/MPa	*E*/GPa	*μ*	*φ*/(°)	*c*/MPa
1	0.0130	0.300	0.0027	0.20	0.01255	0.3001	0.002710	0.1991
2	0.0130	0.325	0.0033	0.45	0.01434	0.3349	0.003310	0.4246
3	0.0130	0.350	0.0039	0.70	0.01231	0.3485	0.003919	0.7002
4	0.0365	0.300	0.0033	0.70	0.03577	0.2945	0.003325	0.7004
5	0.0365	0.325	0.0039	0.20	0.04477	0.3273	0.003561	0.3097
6	0.0365	0.350	0.0027	0.45	0.03810	0.3448	0.002668	0.4696
7	0.0600	0.300	0.0039	0.45	0.06053	0.3033	0.003864	0.4498
8	0.0600	0.325	0.0027	0.70	0.05486	0.3296	0.003062	0.6121
9	0.0600	0.350	0.0033	0.20	0.05526	0.3420	0.003311	0.1909
